# Plasma Levels of Inter-α Inhibitor Proteins in Children with Acute Dengue Virus Infection

**DOI:** 10.1371/journal.pone.0009967

**Published:** 2010-04-06

**Authors:** Penelope Koraka, Yow-Pin Lim, Michael D. Shin, Tatty E. Setiati, Albert T. A. Mairuhu, Eric C. M. van Gorp, Augustinus Soemantri, Albert D. M. E. Osterhaus, Byron E. E. Martina

**Affiliations:** 1 Department of Virology, Erasmus Medical Center, Rotterdam, The Netherlands; 2 ProThera Biologics, Inc., East Providence, Rhode Island, United States of America; 3 Pediatric Department, Dr. Kariadi Hospital, Semarang, Indonesia; 4 Department of Internal Medicine, Slotervaart Hospital, Amsterdam, The Netherlands; 5 Division of Thrombosis and Haemostasis, Beth Israel Deaconess Medical Center, Boston, Massachusetts, United States of America; Singapore Institute for Clinical Sciences, Singapore

## Abstract

**Background:**

Inter-α inhibitor proteins (IaIp) belong to a family of protease inhibitors that are involved in the haemostatic and the vascular system. Dengue viruses (DENV) infections are characterized by coagulopathy and increased vascular permeability. In this study we measured the concentration of IaIp during DENV infections and evaluated its potential as a biomarker.

**Methods and Findings:**

Concentrations of IaIp were measured in patients with acute DENV infections using a quantitative, competitive enzyme linked immunoassay. Concentrations of IaIp measured in pediatric patients suffering from severe DENV infections were significantly lower than in healthy controls.

**Conclusions:**

This is the first report to demonstrate changes in concentration of IaIp during viral infections. The data also highlight the potential of IaIp as a biological marker for severity of DENV infections.

## Introduction

Dengue viruses (DENV) are members of the family *Flaviviridae*, genus *Flavivirus*, and are among the most widely distributed arboviruses world wide that are estimated to infect more than 50 million people annually. DENV infection is usually asymptomatic or may lead to uncomplicated “flu-like” disease with fever, myalgia, arthralgia and rash. At the time of defervescence up to 5% of cases may develop dengue hemorrhagic fever and/or dengue shock syndrome (DHF/DSS). Mortality rates are usually around 1%. However, during large outbreaks of DENV infections if supportive intensive care of the patients is not applied promptly these figure can be much higher. Although the epidemiology is changing, DHF/DSS is still mainly a disease of children, being the leading cause of hospitalization and death among children in South East Asia.

Inter-α inhibitor proteins (IaIp) belong to a family of structurally related serine protease inhibitors that are naturally and abundantly found in human plasma. The major forms in human plasma are the inter-α inhibitor, consisting of two heavy chains and a light chain called bikunin, and the pre-α inhibitor, consisting of one heavy chain and a light-bikunin chain [1]. The physiological functions of these proteins have only recently begun to be revealed. Members of the IaIp family have been involved in physiological as well as pathological activities such as tumor invasion, metastasis and inflammation. For instance, bikunin has been shown to inhibit plasmin, thereby exerting anti-inflammatory and anti-fibrinolytic activities [2].

The potential of IaIp as a diagnostic and prognostic biomarker as well as its therapeutic potential has been demonstrated in bacterial sepsis [3], [4]. However, the role of IaIp during viral infections has not been investigated. In this report we describe the plasma concentrations of IaIp in children with DENV infections and how these concentrations correlate with disease severity.

## Materials and Methods

### Ethics Statement

Written informed consent for blood sampling was obtained from all patients (their parents or legal guardians) included in the studies described in this manuscript. The studies have been approved by the ethics committee of the hospitals involved (Dr. Kariadi Hospital, Semarang, Indonesia and Sophia Children's Hospital, Rotterdam, The Netherlands).

Children infected with DENV were selected from a longitudinal cohort study (n = 200) conducted in Dr. Kariadi Hospital, Semarang Indonesia [5]. Plasma samples were collected on day of hospital admission (day 0), as well as 7 and 30 days later. Day 0 (D0) samples were used to confirm DENV infection by a real time RT-PCR assay. Samples from healthy individuals (n = 37) matching demography and age of the DENV patients were used to determine baseline values of IaIp. These healthy control samples were collected from healthy children that were enrolled in a prospective cohort study (unpublished data) and resided in the same areas as the DENV patients (Semarang, Indonesia), have the same genetic background (Javanese, Indonesian) and matching age and gender. No DENV-specific antibodies were detected in these samples. Samples from 12 children infected with respiratory syncytial virus (RSV), collected on day of admission to hospital (D0) and 30 days later were used for comparison. The characteristics of these patients have been described elsewhere [6].

Concentrations of IaIp in plasma samples were measured with a quantitative competitive enzyme linked immunoassay with a monoclonal antibody against human IaIp (MAb 69.26) as previously described [4]. All the samples were analyzed in a blinded fashion. The non-parametric Mann-Whitney test (GraphPad version 4) was used for statistical evaluation of the IaIp concentrations in the respective groups of patients. Analyses of correlations were done with the Spearman's rho test using SPSS version 11.0.1.

## Results

### Patient's characteristics

In order to exclude patients with clinical picture similar to DENV of other aetiology, the criteria for patient selection was confirmation of DENV infection by demonstrating viral RNA in the D0 samples and complete sampling procedure, i.e. availability of plasma samples from D0, D7 and D30. Based on these criteria, 33 patients were selected to measure the levels of IaIp out of the 200 patients included in the cohort study (Table 1). The cohort consisted of 14 males, 19 females and the median age of the patients was 8 years (range 3–14 years). Seven patients were classified with DF, 15 with DHF, and 11 with DSS according to the WHO criteria [7]. Based on the serological profile of the patients (IgM/IgG ratio) 5 patients could be classified as primary DENV infections whereas the remaining 28 patients had a secondary DENV infection. DENV 1 and 3 were the predominant serotypes (n = 14 and n = 13, respectively), while DENV-2 and DENV-4 were only detected in a few patients (n = 4 and n = 2 respectively), suggesting that all four serotypes of DENV were circulating in Semarang at the time of sampling.

**Table 1:** Characteristics of patients included in the study.

Characteristics	DF patients n = 7	DHF patients n = 15	DSS patients n = 11	RSV patients n = 12	Healthy controls n = 37
Age range (median)	3–14 (12) years	4–13 (10) years	5–12 (6) years	2–6 (4) months	3–6 (4.3) years
Gender, (male n)	4	6	4	7	19
Origin	Indonesia	Indonesia	Indonesia	The Netherlands	Indonesia
Duration of fever range (median)	2–4 (3) days	3–6 (4) days	2–5 (4) days	1–5 (3) days	n.a.
Hematocryte % range (median)*	33–43 (37.8)	33–53 (39.3)	32–51 (41)	n.a.	n.a.
Platelet count cells x 10^3^/mm^3^ * (median)	57–186 (106)	14–207 (66)	10–90 (43)	n.a.	n.a.
Albumin g/l range (median) *	2.0–4.2 (3.7)	1.9–4.3 (3.60	1.0–3.8 (3.2)	n.a.	n.a.
AT III % range (median) *	51–110 (85)	75–120 (97)	50–110 (51)	n.a.	n.a.
APTT sec range (median) *	39–76 (48)	38–84 (53)	49–120 (68)	n.a.	n.a.

*on time of admission.

n.a.: not applicable.

### Concentration of IaIp in acute viral infections

**Figure 1:**
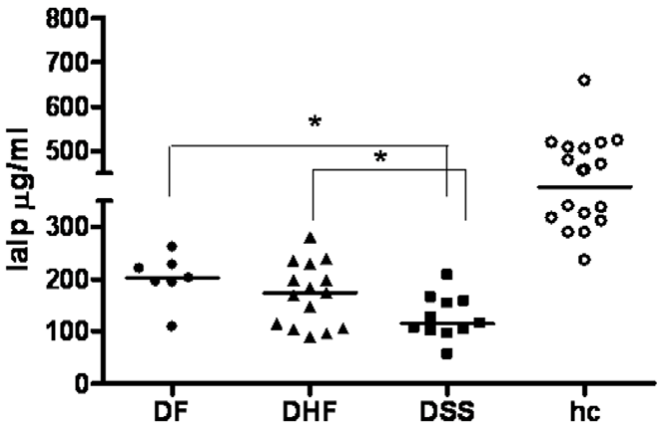
Concentration of IaIp in patients with varying DENV disease severity (day of admission to the hospital) and in healthy controls (hc). *: statistically significant differences with the Mann-Whitney test. Straight lines represent the median.

First, we measured IaIp levels in healthy young children to establish baseline level (median 393 µg/ml, range 236–658 µg/ml) in the study population (Figure 1). Next, we measured the levels of this molecule in plasma from patients with acute systemic (DENV) and non-systemic (RSV) viral infections upon hospital admission. Patients of both groups required hospitalization, indicating that these infections were severe. Both the DENV (median 165, range 56–281 µg/ml) and RSV (median 217, range 145–553 µg/ml) patient groups had significantly lower levels of IaIp on D0 compared to the healthy control group (P<0.0001 and P<0.0001 respectively).

### Concentration of IaIp in DENV infections correlate with disease severity

**Figure 2:**
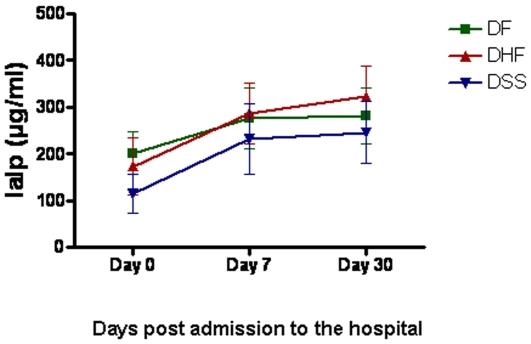
Concentration of IaIp in DENV infected patients with varying disease severity on different time points after infection. Data are depicted as mean ± standard deviation.

Several mediators involved in hemostasis have been associated with DENV-associated disease severity. Therefore, we sought to investigate the association of IaIp concentration with development of DHF and/or DSS. Patients experiencing mild DF, had similar levels of IaIp as patients with acute RSV (D0 median 202, range 109–261µg/ml, P = 0.5867). It is worth noting that the patient in this group, with the lowest concentration of IaIp was classified with “severe DF” i.e. experienced hemorrhagic manifestations but did not meet all criteria of WHO to be classified as DHF grade I-IV. Patients experiencing hemorrhagic fever (DHF grades I-IV) had significantly lower levels of IaIp on D0, compared to patients with mild DF (median 151, range 56–281 µg/ml, P = 0.0443), whereas patients with DSS had the lowest levels of IaIp (median 112, range 56–208 µg/ml) compared to DF, DHF-I/II, and RSV groups (P = 0.0028, P = 0.0461, P = 0.0006 respectively). As shown in figure 2, clinical recovery of DHF patients (grades I–IV) was associated with a rapid and significant increase of IaIp within seven days post admission (median 261, range 152–434 µg/ml, P<0.0001). By D30 of follow up the levels of IaIp between patients recovering from DF (median 282, range 236–414 µg/ml), and DHF grades I–IV (median 283, range 179–414 µg/ml) were similar (P = 0.6066), but lower than the level of IaIp in patients recovering from RSV (median 333; range 205–462 µg/ml). However, the concentrations did not reach levels measured in the healthy controls (P<0.0001), suggesting different normalization kinetics of this molecule during DENV infections compared to RSV infection.

Clinical records and laboratory findings from the day of admission of the DENV infected patients were used to look for association of IaIp and clinical and laboratory markers of disease severity (Table 2). Circulating concentrations of IaIp correlated inversely with disease severity (P = 0.041), ascites (P = 0.010), and activated partial thromboplastin time (aPTT, P = 0.001). In addition there was a correlation of IaIp with thrombocytopenia (P = 0.002), anti-thrombin III (ATIII, P = 0.024) and albumin levels (P = 0.018).

**Table 2:** Correlation of IaIp with clinical and laboratory markers of DENV disease severity measured on day of admission.

Spearman's rho IaIp	severity	Pleural effusion	ascites	Platelet count	Hematocryte	ATIII (%)	aPTT (sec)	Albumin (g/l)
Correlation coefficient	−0.357	−0.248	−0.443	0.514	0.03913	0.492	−0.617	0.415
P value	0.041	0.163	0.010	0.002	0.8289	0.024	0.001	0.018
N	33	33	33	33	33	21	25	32

Significant P values are underlined.

ATIII: anti-thrombin III, aPTT: activated partial thromboplastin time.

## Discussion

This study is the first one to demonstrate that IaIp is significantly decreased during acute viral infections. Our data showed that both a systemic (DENV) and a localized (RSV) viral infection can affect the concentration of IaIp, with a more pronounced effect measured in patients with DSS. Several studies have shown that mediators and other soluble factors (including antibodies and cytokines) differ significantly between DHF and DSS groups [8]–[10].

Plasma levels of IaIp have been shown to be reduced in neonatal and adult bacterial-induced sepsis and the levels correlate inversely with mortality in adult patients with sepsis, prompting investigation of its potential as a prognostic or diagnostic marker [3], [4] as well as its therapeutic potential for sepsis and anthrax intoxication [11], [12]. Fatal outcome of DSS can be significantly reduced when timely supportive treatment and plasma replacement is given to the patients. In this respect, a biomarker with diagnostic/prognostic potential is of paramount importance to assist supportive treatment. In this study we report significant correlations of IaIp and several markers of DENV disease severity, especially thrombocytopenia, a hallmark of severe DENV induced disease (Table 2). These data warrant further studies, designed to elucidate the prognostic potential of IaIp. In this study, we investigated the plasma levels of IaIp in patients that were severely ill and required hospitalization. Studies should be designed in the future to include infected patients prospectively in order to understand the kinetics and prognostic value of IaIp in the early stages of disease (during fever). In light of the therapeutic value of IaIp in sepsis, the potential of this molecule for treatment of DSS should be explored.

The pathogenesis of DENV infections is poorly understood although several studies have concluded that soluble factors and mediators are implicated in the development of DHF/DSS. The significantly decreased concentrations of IaIp in patients with severe disease, in particular in DSS patients, could explain several pathological phenomena seen in DSS. First, the heavy chains of IaIp molecules are known to inhibit activation of both the alternative and classical pathways of the complement system, both *in vitro* and *in vivo* [13]. In this respect, low levels of IaIp could result in the increased levels of C5a often seen in DHF/DSS patients [14]. Second, IaIp inhibit plasmin via its light chain bikunin [2]. Plasmin has a major role in the fibrinolytic system, as an enzyme that degrades fibrin. Impaired fibrinolysis is a hallmark of DSS [10]. In this report, IaIp levels were found to be associated with coagulation (aPTT) and anti-coagulation (ATIII) factors (Table 2), however more studies are needed in order to elucidate the possible role of IaIp in the pathogenesis of DENV infections. Third, IaIp are synthesized and assembled in the liver and low plasma levels of these molecules may reflect liver involvement.

Taken together, this is the first study to highlight the importance of IaIp in acute viral infections and provide an incentive to study the diagnostic and prognostic role of IaIp during acute DENV infections. Our cohort was carefully chosen to include only patients with confirmed DENV infection as demonstrated by the presence of viral RNA in acute phase plasma of the patients. Since little is known about the epidemiology of other viral infections in that region with similar clinical presentation to dengue, we chose to study only the subjects from which DENV viral RNA could be recovered. As a result, our cohort was rather small and therefore other relevant questions such as correlation of IaIp levels and primary or secondary DENV infections could not be addressed. Determination of IaIp concentrations in larger cohorts of patients with different genetic backgrounds, in combination with measurement of other biological markers, such as liver enzymes, are necessary to better understand the role of IaIp in the pathogenesis of DENV infections. Studies are ongoing to address these issues.
